# Effects of a phonics-integrated music rhythm intervention on reading fluency and accuracy with children

**DOI:** 10.3389/fnhum.2025.1636278

**Published:** 2025-07-23

**Authors:** Laura Dees, Patrick K. Cooper

**Affiliations:** Herbert and Nicole Wertheim School of Music, Florida International University, Miami, FL, United States

**Keywords:** musical training, rhythm, literacy, children, reading, phonological awareness, phonemes, music intervention

## Abstract

It is hypothesized that increasing literacy skills such as reading rate (fluency) and reading accuracy with children is possible through intentionally developed interventions focused on musical memory, timing, and production (e.g., clapping to a steady beat). Previous studies have found these effects to be significant with third-grade students, an age when school curriculum emphasizes literacy development. Using a quasi-experimental pretest-posttest design, we tested the effects of a musical intervention integrating English Language Arts (ELA) content from the Fundations program on rhythm and literacy skills with third-grade students aged eight to 10 (*N* = 164). Over 10 weeks, both control and experimental groups attended their regular music class, with the experimental group receiving a higher proportion of class time devoted to building rhythm skills. The most significant training effects were observed in rhythm growth scores: the experimental group showed greater pretest-posttest growth in rhythm skills (*d* = 0.57, *p* < 0.001). Correlations between rhythm and literacy skills were significant at both pretest-posttest time points (*ρ* = 0.20–0.35, *p* < 0.01). However, correlations between growth in rhythm and growth in literacy were not significant. Additionally, no significant differences in literacy growth were found between groups. While these results reinforce the cross-sectional links between rhythm and literacy skills, there was no evidence of transfer from musical growth to literacy growth. These results have practical significance by reporting a lack of training effects despite strong cross-sectional correlations and by discussing methodological considerations for evaluating potential causal effects of music training on academic outcomes.

## Introduction

A growing number of researchers have investigated the potential of music training to increase cognitive ability (Miendlarzewska and Trost, 2014; Cooper, 2019). This foundational concept has led to researchers studying the ability of musical interventions designed to increase cognitive skills to transfer to realized academic improvement (Sala and Gobet, 2017) in domains such as mathematics (Cheek and Smith, 1999) and reading (Anvari et al., 2002). These academic subjects are areas of interest due to the challenges some children experience when trying to learn foundational domain-specific skills. For example, researchers have found that “a substantial proportion of children underachieve in reading” and that these difficulties carry into adulthood (Long, 2014, p. 107). It has been hypothesized that musical development with children, specifically in the area of rhythmic ability, may lead to an increase in reading ability due to the overlapping skills needed to perform music and to be a successful reader, such as the ability to hear and decipher patterns akin to the rhythmic flow of speech and the activation of working memory (Tierney and Kraus, 2013; Cohrdes et al., 2016). The ability to read with flow is known as reading rate or fluency, while the ability to be accurate when reading out loud requires the activation of working memory. Fluency and accuracy are critical skills for reading ability (Kim, 2015).

Researchers have specifically studied the links between rhythmic ability and literacy skills. White and Wesolowski (2019) suggested that “rhythmic ability is strongly correlated to phonological awareness in young children” (p. 2). Bhide et al. (2013) concluded that “giving children rhythmic training and linking non-linguistic rhythms to rhythms in language has a positive effect on literacy acquisition and phonological skills” (p. 120). The results of Long (2014) were used to suggest that focusing on developing multiple rhythmic skills with children not only promoted self-perceived changes in reading, but “largely increased reading rate and comprehension” (p. 115). Despite finding positive links in some studies, a review of the effects of music training on five different reading subskills did not result in “compelling evidence that proficient reading ability and musical training are linked” (Tierney and Kraus, 2013, p. 22). However, a meta-analysis on the ability of music training to enhance literacy skills found small positive effects across nine studies with over 900 participants (Gordon et al., 2015). Potential issues when evaluating results included the interventions being too brief to yield conclusive results (White and Wesolowski, 2019) or finding positive results as a result of the intervention for some reading subskills but not all subskills that were evaluated (e.g., Banai and Ahissar, 2013; Cogo-Moreira et al., 2013).

When looking for any type of transfer between rhythmic growth and literacy growth, it is important to understand how a rhythm intervention can be designed to support literacy development. A rhythm intervention is a type of musical training specifically devoted to performance and awareness of steady beat and patterns, absent of melodic or instrumental training. Common practices include stomping, clapping, and chanting words to steady beats or pulses. Note reading assessments or sight identification may be included in addition to aural practices (Gordon et al., 2015). Interventions are designed to increase awareness of metrical structures, which is hypothesized to be beneficial to the development of phonological awareness, a key component of literacy (Bhide et al., 2013). Some researchers have compartmentalized rhythmic ability as different constructs with slightly different labels (Table 1). Among the similar skills evaluated are the ability to perform different rhythms (complexity), perform them “in time” or “on the beat,” and to use auditory and/or visual memory to assist with performance.

**Table 1:** Three studies with discrete rhythmic skill evaluations.

Study	Skill 1	Skill 2	Skill 3
White and Wesolowski (2019)	Rhythm production	Steady beat ability	Rhythm discrimination
Zanto et al. (2024)	Rhythm-playing complexity	Metrical/timing complexity	Rhythmic memory
Bhide et al. (2013)		Steady beat ability	Rhythm and tempo discrimination

There appears to be no recognizable difference for effects between traditional and digital methods of delivery for a rhythm intervention. White and Wesolowski (2019) used traditional rhythm games found in an American music classroom (e.g., call-and-response playing, clapping) while Zanto et al. (2024) used a digital music game. Both studies resulted in positive effects. White and Wesolowski (2019) found positive growth in oral reading fluency from the intervention while Zanto et al. (2024) concluded that the significant growth in fluency in their study was a result of changes in sensorimotor synchronization from playing the game. Hallam (2018) used an intervention program called Rhythm for Reading with children already deemed to have reading difficulties. After 10 weeks of 10-minute sessions focused on stomping, clapping, and counting along with a notated chart, the experimental group outscored the control group in reading accuracy and comprehension. Bhide et al. (2013) used a digital intervention (GraphoGame) over 2 months on children with pre-screened reading difficulties and found strong correlations between improvement in the rhythm interventions and improvement in the reading assessments.

When analyzing the literature, there is evidence to support the theory that successfully developing certain rhythmic skills with children (e.g., playing/production, timing/steady beat awareness, memory) may lead to increased literacy skills due to the similarities in flow, awareness, memory, and timing between music and reading (Schlaggar and McCandliss, 2007). However, the evidence is not always conclusive. Some studies resulted in significant cross-sectional correlations between rhythmic ability and literacy skills, but without comparing growth in one domain to growth in the other (Cohrdes et al., 2016; Steinbrink et al., 2019; Kertész and Honbolygó, 2021), or without reporting musical growth as a result of the musical intervention (Hallam, 2018). Some studies have quite small sample sizes, which provide excellent trends from which to build future research, but are limited in generalizability (e.g., Bhide et al., 2013). Time is likely an important consideration for implementing an intervention to be used with children in schools, as instructional time for music classes may be limited. Some studies found positive results in as little time as 60-min of training over 6 weeks (Long, 2014), 100-min of training over 10 weeks (Hallam, 2018), and around 5 h of training over 7 weeks (White and Wesolowski, 2019). Conversely, results of meta-analyses suggested much longer periods of training are needed for training effects to be significant (e.g., Gordon et al., 2015; Ahokas et al., 2025). There exists a need for more studies with experimental designs which compare growth in music to growth in literacy with school-aged children.

## Methods and materials

To assess the theory of growth in rhythmic ability enhancing growth in literacy skills with young children, the following research questions guided the study:

1. To what extent does a rhythm intervention lead to musical growth with third-grade students?
2. To what extent does a rhythm intervention lead to literacy growth with third-grade students?
3. To what extent do rhythm skills predict literacy skills?

### Study design

This study was designed as a quasi-experimental trial with pretest-posttest data collection from an experimental group and a control group (Table 2). Two waves of data were collected at the same two schools with different groups of third-grade children (Wave 1 = 2023–2024 school year, Wave 2 = 2024–2025 school year). The students across both waves were the same age during data collection, had the same music teacher both years, and both schools had the same third-grade teachers each year. The music teacher delivered the intervention. There were no significant differences on pre-test measurements between Wave one and Wave two and so they were combined for analysis purposes in subsequent analyses. The pre-test data for all participants and measurements were collected in August, 2023/24 (Time Point 0). The intervention for the experimental groups ran from October, 2023/24 to December 2023/24 (Intervention Period). The post-test data for all participants and measures were collected in January 2024/25 (Time Point 1).

**Table 2:** Study design for data collection.

All participants (*N* = 164)	Early August (time point 0)	August to December (intervention period)	Late January (time point 1)
Wave 1, 2023–2024 Wave 1 Exp (*N* = 40) Wave 1 Con (*N* = 38)	All participants (*N* = 164)	Experimental group (*N* = 82) - Regular music class - Fundations-integrated intervention for 10 weeks (October to December)	All participants (*N* = 164)
	Fall rhythm pre-test		Winter rhythm pre-test
Wave 2, 2024–2025 Wave 2 Exp (*N* = 42) Wave 2 Con (*N* = 44)	Fall MAP pre-tests - Fluency level - Accuracy level - Accuracy percentage	Control group (*N* = 82) - Regular music class only	Winter MAP post-tests - Fluency level - Accuracy level - Accuracy percentage

Each intervention lasted 10–15 min and each experimental group received 20 sessions over 10 consecutive weeks (two sessions per week). This amount of training time is similar to other studies which took place at schools with young children (e.g., Hallam, 2018) and had the intervention delivered by the participants’ regular classroom music teacher (e.g., Long, 2014; White and Wesolowski, 2019). While potentially increasing ecological validity by having the classroom music teacher deliver the intervention during regularly scheduled class time, this design does present issues of time constraints which are discussed in the implications section of this paper. The protocol for the intervention is described in more detail under the Measures section of this paper. The post-test data collection for all participants and measurements occurred four to 6 weeks after the conclusion of the intervention period. As it was hypothesized that the intervention may be beneficial to students, the control group received the full Fundations-integrated rhythm intervention after the conclusion of data collection.

### Participants

The participants in this study were (a) aged eight to 10 years old and attended a public elementary school in the United States, (b) could be any gender identity, (c) could be any race, and (d) had been in a general music class the previous year. Students participated with their entire third-grade class. Two schools participated, one in an urban setting and one in a suburban setting. One third-grade class from each school was designated a control group and one third-grade class from each school was designated an experimental group. In both schools, students had 45-min general music classes on a bi-weekly basis starting in kindergarten. The curriculum for the regular third-grade music class included a variety of musical experiences including rhythm skill building, instrumental technique, vocal instruction, Orff instruction, composition activities, and improvisation games. In this school district, grade three is the final year in which students study phonics using the Fundations curriculum.

### Measures

#### Dependent variables

The dependent variables in this study were a rhythm skills test designed by the music teacher at the two schools and three subsections of the Northwest Evaluation Association (NWEA) Measure of Academic Progress (MAP) assessments. The three subsections were Fluency Level, Accuracy Level, and Accuracy Percentage. Fluency is largely a phonological measurement while accuracy requires both phonological and phonemic awareness.

#### Rhythm skills

Scores for the rhythm skills test were collected by the students’ music teacher. The instrument used to measure rhythm skills was created by the music teacher at the schools and included a memory task, notation-based production task, and aural rhythm timing-complexity task. Questions one through seven were multiple-choice options where students matched standard notation symbols to duration values (memory task). Questions eight and nine (worth two points each) had students compose their own rhythms using standard notation which equaled one measure of 4/4 time on a blank staff (notation-based production task). Questions 10 and 11 (worth two points each) had students dictate aural patterns using standard notation based on the teacher performing rhythms as syllables on a blank staff (i.e., Kodály-style ta, ti-ti; aural rhythm timing-complexity task).

#### NWEA MAP scores

NWEA MAP scores were collected in the students’ third-grade classrooms under the supervision of their third-grade teachers. To collect NWEA MAP scores, students read several passages out loud into a microphone. Their readings were transcribed and scored for fluency and accuracy by the NWEA software. Fluency level is an ordinal measurement of oral reading rate and is labeled as below grade level (coded as 1), approaching grade level (coded as 2), meets grade level (coded as 3), or exceeds grade level (coded as 4). Accuracy level is an ordinal measurement of correct words read at certain difficulty levels during the passages and is labeled as below grade level (coded as 1), approaching grade level (coded as 2), meets grade level (coded as 3), or exceeds grade level (coded as 4). Accuracy percentage is a more generic measurement of correct words read regardless of difficulty level.

EdMetric examined the reliability of NWEA MAP data from 2016 to 2017 via test–retest reliability, marginal reliability, and score precision. They concluded the standard error of measurement was low due to an adaptive test algorithm, which changes according to student need. All reliability scores were consistent and in appropriate ranges. On average, “97.4% of the items were aligned to the (Common Core State Standards) CCSS across all grades and content areas” (Egan and Davidson, 2017).

#### Rhythm intervention (independent variable)

The control group and experimental group both attended their regular 45-min, bi-weekly music class during the intervention period, the curriculum for which was described above. The experimental group received 20 intervention sessions during their regular music class from October to December. This means the experimental group did not receive more music training, but rather the time spent in class was more heavily allocated toward rhythm growth by implementing the Fundations-integrated rhythm intervention. This feature may be important to highlight as previous researchers using a similar protocol have suggested it is necessary to distinguish between what occurs during a rhythm training intervention and during a general music class (Ahokas et al., 2024). The activities in the intervention supported building phonemic awareness by breaking down smaller sounds within words and sentences to steady beats. The activities in the intervention supported building phonological awareness by using rhyming sentences and segmenting words to rhythmic patterns. These are not typical activities in a traditional general music class. A feature of the present study was to evaluate the effects of training activities specifically for rhythm on literacy, rather than taking a more constructivist approach to learning rhythm through varied musical experiences (e.g., singing, playing instruments).

The intervention occurred in the first 10–15 min of each regular music class. The intervention started with clapping and speaking syllables (e.g., ta, ti-ti, etc.) out loud with a rhythm notation video from the *YouTube* channel “Visual Musical Minds” (Walby, 2023). The selected videos started with note values commonly found in the third-grade music curriculum such as eighth notes (quavers), quarter notes (crotchets), and rests, with progressively more challenging timings added over each session, culminating with sixteenth notes (semiquavers), dotted quarter notes (dotted quavers), dotted half notes (dotted minims), and syncopated rhythms.

In the next activity, students spoke sentences taken directly from Units One and Two of their Fundations curriculum, which was used by their third-grade teachers to help prepare them for their NWEA MAP tests. Fundations is a structured literacy program that is “sequential, systematic, and cumulative” (Curriculum - Wilson Language Training Corporation, 2023). According to this curriculum, students should be able to use knowledge of language and its conventions when reading and be progressing toward reading fluently by the end of grade three. The sentences taken from the Fundations units included phrases such as “the drummer tapped on the drums again,” and increased in difficulty throughout the intervention period. First, students would read the sentences as if they were reading them from a passage. Then, students would speak the sentences rhythmically over a background track with beats, both by echoing the teacher and by themselves.

### Statistical analyses

The following analyses were planned to answer each research question.

Question #1: To what extent does a rhythm intervention lead to musical growth with third-grade students? Analysis: An analysis of variance (ANOVA) will be conducted on the growth scores for rhythm skills between the control and experimental group to estimate the statistical and practical effects of the intervention on rhythm skills.

Question #2: To what extent does a rhythm intervention lead to literacy growth with third-grade students? Analysis: An analysis of variance (ANOVA) will be conducted on the growth scores for NWEA MAP fluency level, accuracy level, and accuracy percentage between the control and experimental group to estimate the statistical and practical effects of the intervention on measurements of literacy skills.

Question #3: To what extent do rhythm skills predict literacy skills? Two analyses: (1) Spearman-rank correlations between the dependent variables at pre- and post-test time points will be used to assess cross-sectional relationships between rhythm and literacy. (2) Spearman-rank correlations for growth on the dependent variables will be used to further assess the effects of rhythm growth on literacy growth.

## Results

To understand the need for covariates, an analysis of variance (ANOVA) was conducted on rhythm, MAP-fluency, MAP-accuracy percentage, and MAP-accuracy level pre-test measurements. The results of the ANOVA were not significant (Table 3), suggesting there were no differences between the control group and experimental group on these measurements. Therefore, pre-test scores were not used as covariates in any subsequent analyses.

**Table 3:** Means, standard deviations, and *F-*test significance levels for rhythm and literacy skills.

	Rhythm skills pre-test	Rhythm skills post-test	Rhythm skills growth*
Control (*N* = 82)	6.98 (3.45)	8.13 (3.16)	1.16 (2.67)
Exp (*N* = 82)	6.06 (3.59)	8.60 (3.22)	2.54 (2.15)
Group difference	*n.s.*	*n.s.*	*p < 0.001, *d* = 0.57

	Fluency level pre-test	Fluency level post-test	Fluency level growth
Control (*N* = 82)	1.77 (0.43)	1.80 (0.40)	0.04 (0.40)
Exp (*N* = 82)	1.71 (0.46)	1.77 (0.43)	0.06 (0.40)
Group difference	*n.s.*	*n.s.*	*n.s.*

	Accuracy % Pre-test	Accuracy % Post-test	Accuracy % growth
Control (*N* = 82)	89.84% (6.39%)	91.90% (5.22%)	2.60% (4.20%)
Exp (*N* = 82)	89.54% (7.41%)	91.68% (6.31%)	2.60% (6.70%)
Group difference	*n.s.*	*n.s.*	*n.s.*

	Accuracy level pre-test	Accuracy level post-test	Accuracy level growth
Control (*N* = 82)	1.89 (0.92)	2.18 (0.93)	0.29 (0.78)
Exp (*N* = 82)	1.94 (0.85)	2.23 (0.87)	0.29 (1.04)
Group difference	*n.s.*	*n.s.*	*n.s.*

To assess growth within a domain, paired samples *t*-tests were used to look for significant differences between pre- and post-test measurements. The within-subjects growth for rhythm skills (*d* = 0.74), accuracy percentage (*d* = 0.32), and accuracy level (*d* = 0.45) were all significant (all *ps* < 0.001), with a trend toward significance for fluency level (*p* = 0.12, *d* = 0.12). This indicates that students generally had higher post-test scores compared to their pre-test scores on the dependent variables.

### Effects of the rhythm intervention on rhythm skills

To analyze the effects of the rhythm intervention on rhythm skills with children, an analysis of variance (ANOVA) was conducted on the post-test and growth scores for the control group and experimental group (Table 3). The results of the ANOVA were not significant for the post-test (*p* < 0.05), which indicates there was not a statistical difference between the control group and experimental group on the post-test. However, the rhythm growth scores between the control group and experimental group were significant with a large effect size, *F*(1, 162) = 13.21, *p* < 0.001, *d* = 0.57. This indicates the experimental group overall had larger growth in their musical rhythm skills as a result of the rhythm intervention than did the control group.

### Effects of the rhythm intervention on literacy skills

To analyze the effects of the rhythm intervention on reading fluency and accuracy with children, an analysis of variance (ANOVA) was conducted on the post-test and growth scores for MAP-fluency, MAP-accuracy percentage, and MAP-accuracy level scores. The results of the ANOVA were not significant for all measures (*ps* < 0.05). This indicates there was not a statistical difference between the control group and experimental group for any measurements of literacy on the post-test or growth scores.

### Rhythm skills as a predictor of literacy skills

It was desired to learn to what extent scores on the rhythm skills test predicted scores for literacy skills at each time point (pre- and post-test) and with growth scores. Spearman-rank correlations were used to analyze the relationships between the dependent variables (Table 4). The results indicated significant correlations between rhythm skills and literacy skills at the pre- and post-test time points.

**Table 4:** Spearman-rank correlations for rhythm and literacy, pre- and post-test scores.

*N* = 164	Rhythm pre	Fluency pre	Acc % pre	Acc lev pre	Rhythm post	Fluency post	Acc % post	Acc lev post
Rhythm Pre	–							
Fluency Pre	0.336** < 0.001							
Acc % Pre	0.217* 0.005	0.506** < 0.001						
Acc lev Pre	0.202* 0.010	0.445** < 0.001	0.935** < 0.001					
Rhythm Post	0.695** < 0.001	0.245* 0.002	0.326** < 0.001	0.289** < 0.001				
Fluency Post	0.297** < 0.001	0.569** < 0.001	0.474** < 0.001	0.392** < 0.001	0.204* 0.009			
Acc % Post	0.344** < 0.001	0.485** < 0.001	0.603** < 0.001	0.536** < 0.001	0.418** < 0.001	0.475** < 0.001		
Acc lev Post	0.349** < 0.001	0.457** < 0.001	0.567** < 0.001	0.500** < 0.001	0.403** < 0.001	0.440** < 0.001	0.952** < 0.001	–

**p < 0.001 (two-tailed), *p < 0.01 (two-tailed).

However, Spearman-rank correlations were not significant between rhythm skills and literacy skills when looking at pretest-posttest growth (Table 5). This could be interpreted to mean that while there appears to be a relationship between rhythm skills and literacy skills, increased musical growth as a result of the rhythm intervention was not the reason for growth in literacy skills for students in this study. The growth in rhythm skills did not correlate with growth in literacy skills.

**Table 5:** Spearman-rank correlations for rhythm and literacy skills, growth scores.

*N* = 164	Rhythm growth	Fluency growth	Accuracy % growth	Accuracy level growth
Rhythm growth	–			
Fluency growth	0.022 0.780			
Accuracy % growth	−0.079 0.316	0.121 0.123		
Accuracy level growth	−0.038 0.626	0.044 0.574	0.718** < 0.001	–

**p < 0.001.

## Discussion

Both groups experienced growth in rhythm and literacy skills over the duration of the study, an effect of Time. In this study, the musical intervention appears to have led to superior growth for the experimental group in rhythm skills, but not higher growth for literacy skills. When looking at pre- and post-test correlations between rhythm and literacy skills, there appears to be strong links between the two domains. However, growth in rhythm skills did not correlate to growth in literacy skills when analyzing the within-subjects data.

### Effects of the rhythm intervention on rhythm skills

Finding superior growth in rhythm skills is a significant finding for the efficacy of the intervention for music education or future research purposes, noting that the experimental group data were compared to an active control group still engaged in a music class. The intervention was unique as it included traditional (e.g., chanting, use of standard notation) and digital components (i.e., use of *YouTube*). Additionally, post-test data were collected four to 6 weeks after the intervention concluded, showing the permanence of the rhythm intervention on rhythm skills, in this study meaning a combination of memory, production, and aural skills tasks. The intervention integrated grade-level phonological (sentences) and phonemic (syllables) awareness pedagogy into a music curriculum, which led to superior growth in rhythm skills for students. This aligns with previous research suggesting there are deep links and potential transfer effects with the development of music skills and language (Cohrdes et al., 2016) and that phonological awareness as a cognitive skill appears to be heavily linked with rhythm processing (Ozernov-Palchik and Patel, 2018; Loui et al., 2011). Time was a significant factor, in that both groups experienced growth in rhythm skills and literacy skills overall. This is similar to previous research that has found Time as the most significant predictor for the development of phonological awareness (Kempert et al., 2016). Documenting and analyzing growth between both domains could be an important methodological feature as more researchers investigate ways to use music as an intervention to build literacy skills with children. Establishing a causal link between rhythm growth and literacy growth would need to entail a linear relationship between the two outcomes, as was attempted with the design of this study.

### Effects of the rhythm intervention on literacy skills

When comparing growth in literacy skills of the experimental group to the active control group also participating in music classes, the effects of the rhythm intervention were not significant in enhancing literacy skills. While perhaps less common in recently published research, these findings do align with some studies that found musical training did not increase literacy skills with children (Kempert et al., 2016). A meta-analysis on the ability of music training to enhance literacy skills (Gordon et al., 2015) resulted in a mean effect size of *d* = 0.20, 95% CI (0.04, 0.20), suggesting the true mean of the effects were somewhere between 0.04 and 0.20, a rather large amount of variance which borders on non-significant. Importantly, this means the 95% prediction interval would contain a negative value, suggesting a 95% chance future studies with similar methods would yield a non-significant or negative effect. This interpretation aligns strongly with the results found in the present study.

A similar study by Ahokas et al. (2024) with students aged six to eight resulted in non-significant differences in literacy growth between an experimental group receiving a rhythm training intervention during their music classes and a control group receiving regular school music classes only. In Ahokas et al. (2024) and in the present study, the within-subjects analysis showed literacy growth for both groups despite a lack of training effects. This may show how an emphasis on literacy skills at the school level during certain ages may positively influence within-subjects data or make between-groups comparisons less likely to be significant when studying literacy during this developmental stage. However, the results from the present study may also reinforce a trend in research that longer intervention periods than our training protocol are needed for training effects to be significant (Gordon et al., 2015; Ahokas et al., 2025).

### Rhythm skills as a predictor of literacy skills

There were strong significant correlations between rhythm skills and all three measurements of literacy skills when conducting a within-subjects analysis at two time points (pre- and post-test). Finding correlations between the two domains is consistent with previous studies (e.g., Lamb and Gregory, 1993; Anvari et al., 2002; Peynircioglu et al., 2002; Dellatolas et al., 2009; Chobert et al., 2011; Tierney and Kraus, 2013, as cited in, Jantzen, 2017). Additionally, correlations between rhythm skills and literacy skills in this study (*ρ* = 0.20–0.35, *p* < 0.01) were very similar to another study with third-grade students (Steinbrink et al., 2019), from a different country speaking a different language (Germany/German), which correlated phonemic awareness with rhythm perception (0.24, *p* < 0.01) and rhythm reproduction (0.38, *p* < 0.05). This reinforces the idea that rhythm skills have overlap with literacy skills (e.g., David et al., 2006; Gordon et al., 2015; Bonacina et al., 2018; Politimou et al., 2019, as cited in Kertész and Honbolygó, 2021).

The correlations between rhythm and literacy skills growth were not significant. Both groups experienced growth in rhythm skills and literacy skills, however, there was no evidence to suggest that there was a causal relationship between growth in the two domains. The reason for within-subjects literacy growth cannot be attributed to musical growth in this study, which is further supported by the non-significant analysis of variance (ANOVA) between the experimental group and control group post-test scores. This contradicts previous research cited in the introduction which found significant growth between groups on literacy growth as a result of a shorter musical intervention (Hallam, 2018; White and Wesolowski, 2019; Zanto et al., 2024). While the results of these studies differ from ours by finding significant between-groups differences on post-tests, they did not compare growth in rhythm to growth in literacy skills.

### Limitations and future research

Overall, the results of this study align with previous research by demonstrating a cross-sectional relationship between rhythm skills and literacy skills while failing to find a statistically significant relationship between growth in rhythm skills and growth in literacy skills. This study used a researcher-developed protocol and measurement of rhythm skills. While the protocol was developed through use of theory, this could be a limitation as the protocol and measurement were previously untested. It could be worthwhile to add a tapping task as a dependent variable, recognizing that tapping has been shown to be highly correlated with the ability to read text (Goswami et al., 2002; Thomson and Goswami, 2008; Flaugnacco et al., 2015).

We ultimately desired a close-to-practice design (Wyse et al., 2018) similar to what was described in Ahokas et al. (2024). As this study took place at schools, it was not possible to control certain extraneous variables, such as interruptions to regular class schedules and absences from students. Additionally, it was reported that some classroom teachers of the experimental groups would occasionally do the musical games and chants from the music class with their students. It is unclear to what extent these impromptu or spontaneous music making sessions influenced musical growth in the experimental group. It would be ideal to use random selection with participants, perhaps in a pullout or after-school setting, to try to eliminate additional exposure to the treatment from other teachers aware of the study. Using the classroom music teacher to deliver the intervention was ideal for ecological validity.

Another limitation is that the researchers were unable to match students on any other variables (e.g., mother’s education level) which are known to be highly accurate predictors of reading accuracy (Flaugnacco et al., 2015). Additionally, students in this study were not pre-screened for reading difficulties (e.g., Bhide et al., 2013; Hallam, 2018). It would be preferred in future studies to be able to collect more background information (e.g., SES) and measurements of non-literacy skills (e.g., IQ) with participants to be able to randomly assign them to matched groups and to evaluate the need for more control variables in the analyses.

The amount of training time received by the participants may have also influenced the results of the study. Both groups received 30 h of general music classes total during the intervention period. The classes occurred in 45-min instructional periods, twice per week. Participants in the experimental group received around 4.25 h of exposure to the rhythm intervention during their bi-weekly general music class. Ahokas et al. (2025) suggested 90–120-min sessions twice per week may be needed for training effects to be significant. Previous studies with similar hours of exposure to the intervention (Gromko, 2005, 6.5 h; Bolduc and Lefebvre, 2012, 6.67 h; Thomson et al., 2013, 3 h) also resulted in non-significant training effects on literacy when analyzed as part of a meta-analysis (Gordon et al., 2015). Interestingly, Moreno et al. (2009) used 60 h of training and did find significant training effects on literacy. This aligns with a conclusion from Gordon et al. (2015) that 50 h of training or more may be needed for training effects to be significant. In the present study, longer periods of instruction were not possible due to the trainings taking place during regularly scheduled class times. When conducting future research with a close-to-practice design, it may be advantageous to increase exposure time by including the rhythm training intervention in every music class for an entire school year.

### Implications

The results from this study do not suggest a causal relationship between growth in rhythm and literacy skills, even though cross-sectional correlations at two Time points were highly significant. This could be an important methodological consideration for future research. It is important to evaluate growth between the two domains. We suggest there is a need for more studies which evaluate correlations or regression models comparing growth across both domains.

The design of this study included evaluating the permanence of the rhythm intervention, as there was a four to 6 week break between the conclusion of the intervention period and all post-test data collection. The benefits of the rhythm intervention were still present weeks later. Both groups experienced regular music classes, which could explain the significant growth by both groups. The additional rhythm growth found in the experimental group echoes the conclusion of Ahokas et al. (2024) that a specific protocol devoted to rhythm development can lead to different results than participation in a general music class only. The rhythm intervention described in this study appears to be a novel method for growing rhythm skills with third-grade students. The idea of integrating a phonics-based curriculum to reinforce musical ideas is one which appears beneficial and could be used in future research and classrooms alike. When teaching reading foundations and fluency, English Language Arts (ELA) teachers often employ clapping, rhythmic speaking, and flow to assist their readers in being successful (Hall and Robinson, 2012). The positive effects of these strategies may be bidirectional, in that including ELA strategies for reading in a music curriculum appears to be worthwhile in assisting the development of musical rhythm skills. More research is needed to untangle the bidirectionality of this theory.

A final consideration is to contemplate how the age of the participants may have influenced the results. Previous studies have found lesser effects with pre-school aged children compared to older students (Kempert et al., 2016) as well as stronger effects when participants are in later stages of language development (Tierney and Kraus, 2013). The participants in this study were not expected to be fluent in reading until the end of their third-grade year, according to the curriculum information provided by their teachers. Based on this theory, it could be possible that the effects would have been more pronounced if participants were slightly further ahead in their reading development overall. The students in this sample were not yet at grade-level fluency according to the MAP scores, which is acceptable, considering they had completed only half of their third-grade school year during post-test data collection. The data in this study were collected at the beginning of the school year and at the midpoint. It would be advantageous in future studies to collect a third round of data at the very end of the school year and to take a longitudinal approach across an additional time point.

## Data Availability

The raw data supporting the conclusions of this article will be made available by the authors, without undue reservation.
