# Correction: Effects of a phonics-integrated music rhythm intervention on reading fluency and accuracy with children

**DOI:** 10.3389/fnhum.2025.1672040

**Published:** 2025-08-18

**Authors:** Laura Dees, Patrick K. Cooper

**Affiliations:** Herbert and Nicole Wertheim School of Music, Florida International University, Miami, FL, United States

**Keywords:** musical training, rhythm, literacy, children, reading, phonological awareness, phonemes, music intervention

The reference for Ahokas et al., 2024 was erroneously written as: Ahokas, J. R., Saarikallio, S., Parviainen, T., and Louhivuori, J. (2024). The training of rhythm skills and executive function: a systematic review. *Ann. N. Y. Acad. Sci*. 1533, 156–168. doi: 10.1111/nyas.15099

It should be Ahokas, J. R., Saarikallio, S., Welch, G., Parviainen, T., and Louhivuori, J. (2024). Rhythm and reading: connecting the training of musical rhythm to the development of literacy skills. *Early Child. Educ. J*. 53, 999–1012. doi: 10.1007/s10643-024-01654-4

The reference for Ahokas et al., 2025 was erroneously written as Ahokas, J. R., Saarikallio, S., Welch, G., Goswami, U., and Parviainen, T. (2025). The Training of Rhythm Skills and Executive Function: A Systematic Review. *Music & Science*. 8:20592043241305922. doi: 10.1177/20592043241305922

It should be Ahokas, J. R., Saarikallio, S., Welch, G., Goswami, U., and Parviainen, T. (2025). The training of rhythm skills and executive function: a systematic review. *Music Sci*. 8:20592043241305922. doi: 10.1177/20592043241305922

The original version of this article has been updated.

